# Structural Characterization of Lignin in Fruits and Stalks of Chinese Quince

**DOI:** 10.3390/molecules22060890

**Published:** 2017-05-27

**Authors:** Hui-Shuang Yin, Hua-Min Liu, Yu-Lan Liu

**Affiliations:** Department of Oil Engineering, College of Food Science and Technology, Henan University of Technology, Zhengzhou 450001, China; hsyinMars@163.com (H.-S.Y.); liuyl7446@163.com (Y.-L.L.)

**Keywords:** Chinese quince, milled wood lignin, ^31^P-NMR, HSQC

## Abstract

Chinese quince (*Chaenomeles sinensis*) is used in food and pharmaceutical products, but it is seldom eaten as a raw fruit due to its astringent, woody flesh. The structural characterization of lignin fractions from Chinese quince was very important to investigate the structure-activity relationships of lignin. In this investigation, to characterize the structure of lignin in Chinese quince fruits, the milled wood lignin sample was isolated from the fruits (FMWL) and the chemical structure of FMWL was investigated by sugar analysis, FT-IR, GPC, pyrolysis-GC/MS analysis, UV spectra analysis, thermogravimetric analysis (TGA), and advanced NMR spectroscopic techniques. In addition, the lignin fraction from the stalk of Chinese quince (SMWL) was also prepared for comparison to obtained more information of lignin structure in the fruits. The results showed that the two lignin fractions isolated from fruit and stalk of Chinese quince exhibited different structural features. The two MWL samples were mainly composed of β-*O*-4 ether bonds, β-5 and β-β′ carbon-carbon linkages in the lignin structural units. Compared to the SMWL, the FMWL fraction had the higher S/G ratio and more carbohydrates linkages. The predominant carbohydrates associated with FMWL and SMWL fractions were glucans-type hemicelluloses and xylan-type hemicelluloses, respectively. Understanding the structure of lignin could give insight into the properties of the lignin and enable the food processing industry to separate lignin more efficiently.

## 1. Introduction

Chinese quince (*Chaenomeles sinensis; Rosaceae*) is native to China, Japan, and Korea in eastern Asia [1]. It is popularly used for treating many diseases such as colds, coughs, beriberi, asthma, and so on, however, as a fruit, it is hard to eat due to its low moisture content, woody flesh, and a strong acidity and astringency because of the higher amount of stone cells and development of lignum [2]. The high amounts of lignin in the fruits limits its use in the food industry and they are often processed as starch syrup, quince tea, quince wine, etc. Recently, many attempts have been made to investigate the antioxidant and antiulcerative properties of Chinese quince extract components such as polyphenols, flavonoids and organic acids [3,4,5]. However, no investigation has been conducted on the structural properties of lignin in Chinese quince.

As a natural and non-toxic phenolic polymer, lignin is widely applied in various areas, because of its structural features. For example, it is a very frequently used feedstock to obtain vanillin as a beverage and food flavouring agent [6]. Lignin isolated from various plants can be also used as an effective free radical scavenger due to its antioxidant properties [7]. It would be of great significance for the utilization of Chinese quince fruits if the lignin could be extracted efficiently during food processing. In addition, the woody hard flesh of Chinese quince may also be explained by the presence of lignin in the raw fruit. Therefore, structural characterization of lignin from Chinese quince is very important, and should be helpful for investigation of the structure-activity relationships of lignin. In addition, more information about the lignin structure and the chemical linkages associated with carbohydrates is beneficial for the separation of the polysaccharides from the fruits.

Lignin is one of the most abundant heterogeneous biopolymers in Nature, constituting up to 1/3 of the plant cell walls [8]. Generally, it is formed by the dehydrogenative polymerization of mainly three phenylpropane units (sinapyl alcohol, coniferyl alcohol, *p*-coumaryl alcohol) linked through different types of carbon-carbon and ether bonds [9]. These different phenylpropane units can give rise to various types of lignin units, known as syringyl (S), guaiacyl (G), and *p*-hydroxyphenyl (H) types [10,11]. The chemical structure and amount of lignin vary among plant types, cell types, and individual cell wall layers. For example, hardwood lignin is mainly made up of G and S-type units, while lignin in softwood is almost exclusively of G-type units, while all the three lignin units are present in the Gramineae (grasses, bamboo) [12]. However, until now, the characteristics of lignin have not yet been completely characterized because of its complex chemical composition and structure. Lignin has been classified into various types such as milled wood lignin, acid-insoluble lignin, alkali lignin based on the different preparation methods used. Among these lignin types, the milled wood lignin (MWL) can be considered as the most representative of native lignin in terms of chemical structure because the preparation process involves only physical modification. Therefore, the MWL is typically used in the investigation of native lignin structures.

To the best of our knowledge, the detailed chemical structure of lignin in Chinese quince fruit has not been investigated until now. More structural information on lignin in Chinese quince fruit is very important for understanding the cell wall structure and the properties of lignin. In this study, the MWL fraction from the fruit of Chinese quince has been extensively investigated for characterization of the chemical structure and composition, as well as the linkages between units. In addition, the MWL fraction from the stalk of Chinese quince was also prepared for comparison to obtained more information about the lignin structure in the fruits. The structural features of the two MWL samples were comparatively investigated by carbohydrate analysis, molecular weight analysis, FT-IR analysis, pyrolysis-GC/MS analysis, thermogravimetric analysis (TGA), and advanced NMR spectroscopic techniques, including ^13^P-NMR spectroscopy and 2D HSQC NMR spectroscopy.

## 2. Results and Discussion

### 2.1. Chemical Composition of Isolated MWL Fractions

The yields and carbohydrate contents of lignin fractions isolated from the fruits and stalks of Chinese quince are given in Table 1. The yields of stalk milled wood lignin (SMWL) and fruit milled wood lignin (FMWL) were 7.3% and 0.8%, respectively. Although most carbohydrates have been removed from the two MWL fractions during purification precess, some amount of carbohydrates may have been remained because of the chemical linkages between carbohydrates and lignin. As can be seen in Table 1, both samples contained small amounts of sugars, amounting to 0.67% and 1.39% for SMWL and FMWL, respectively. It can also be seen that the carbohydrate composition of the two MWL fractions was different. In the FMWL sample, the predominant sugar was glucose, which comprised 58.3% of the total sugars, while cellulose was its major carbohydrate component. The secondary major sugars in FMWL were galactose and mannose, comprising 18.7 and 15.8% of the total sugars, respectively. In addition, trace amounts of arabinose (7.2%) were also observed in FMWL. As for SMWL, its carbohydrate composition was significantly different. The predominant sugar in SMWL was xylose (0.26%), which constituted 38.8% of the total sugars, indicating that the SMWL may contain more linear hemicelluloses (xylans). The secondary major sugar was glucose, which comprised 20.9% of the total sugars; this it was followed by arabinose, galactose and gluconic acid in amounts of 0.10, 0.09 and 0.08%, respectively.

### 2.2. FT-IR Characterization

To determine the structural differences between SMWL and FMWL fractions, the fragments and functional groups of the two samples were investigated by FT-IR. The results are listed in Figure S2 (Supplementary Materials). As shown, the SMWL fraction shows typical lignin signals of woody plants [13]. The signal for the O–H stretching vibration in aliphatic and aromatic OH groups was observed at the broadly-stretched absorption band around 3411 cm^−1^. The band at around 2939 cm^−1^ corresponds to the C–H symmetrical and asymmetrical vibrations in methylene and methyl groups, respectively. The bands at 1608 and 1514 cm^−1^ can be attributed to aromatic skeleton vibration and this signal pattern is typical of lignin. The signal at 1460 cm^−1^ is attributed to C–H deformation combined with aromatic ring vibration, while the weak absorption band at 1363 cm^−1^ is attributed to the aliphatic C–H stretch in phenolic hydroxyl and methyl groups. Correspondingly, the absorption band at 1329 cm^−1^ is most likely due to condensed guaiacyl and syringyl rings breathing with C–O stretching, and the band at 1267 cm^−1^ is related to guaiacyl ring breathing with C=O stretching. It should be noted that the strong band at around 1122 cm^−1^ shows that the lignin in stalk of Chinese quince is of the GS type [14]. As compared with the SMWL fraction, the FMWL showed similar absorption bands and relative intensities in the fingerprint region. This similarity indicated that the structure properties of lignin in fruit and stalk were not significantly different. However, an important difference in the 1737 cm^−1^ band is observed in the FMWL fraction which is attributed to C=O stretching of carbonyl, unconjugated ketone, and ester groups. This was probably because of variation in the chemical structures of the two lignin fractions or in the chemical linkages between lignin and carbohydrates. Another prominent different absorption band at 1227 cm^−1^ in the FMWL spectra is due to the C–C, C–O and C=O stretching. A maximum absorption band at 1124 cm^−1^ was observed in FMWL fraction, which indicated that the lignin in fruit was the same GS type lignin as compared with SMWL.

### 2.3. UV Spectra Analysis

UV spectroscopy was used to investigate the structure of the two MWL fractions because of the ultraviolet absorption of the conjugated carbonyl and aromatic rings. In this investigation, UV spectra in the range from 200 to 600 nm were investigated to indentify the two MWL fractions. A typical maximum absorption peak at around 280 nm is most commonly used in the UV spectroscopic determination of lignin [15]. UV spectra of FMWL and SMWL in water-dioxane solution (1:9, *v/v*) are shown in Figure S3 (Supplementary Materials). Clearly, the two lignin samples showed similar absorption maxima at around 250 and 280 nm. The two lignin fractions show the basic UV spectrum of typical lignin with an absorption band at about 280 nm, which was due to non-conjugated phenolic groups (aromatic rings) of lignin [16]. The striking UV bands appearing at 250 and 253 nm may be attributable to unsaturated bonds and carbonyl groups.

### 2.4. Molecular Weight Analysis

**Figure 1:**
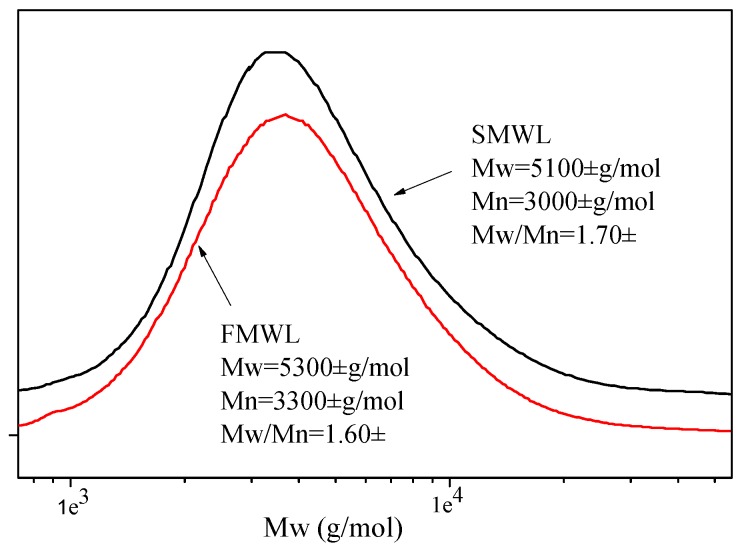
GPC chromatograms of FMWL and SMWL fractions.

The number-average (Mn), weight-average (Mw) molecular weights, and polydispersity (Mw/Mn) of FMWL and SMWL samples are listed in Figure 1. It should be noted that the two MWL fractions were soluble in tetrahydrofuran after derivatization, and this solvent was used as eluant in the present experiment. Figure 1 shows the GPC chromatogram of the two lignin fractions. The chromatograms of SMWL and FMWL showed similar weight-averaged molecular weights of ~5100 and ~5300 g/mol, respectively. In addition, the two lignin samples exhibited relatively narrow distributions of molecular weight, as shown by Mw/Mn < 2. The polydispersity of FMWL (~1.60) was only slightly lower than SMWL (~1.70). A previous investigation showed that accessibility to higher molecular weight lignin fractions was increased as the ball-milling time was prolonged [17]. Longer ball-milling time probably decreases powder size, which would favor the extraction of lignin with higher molecular weight. It can be seen in Figure S3 of Supplementary Materials that diameter of fruit particles was smaller than stalk particles, which favored the bigger molecular weight lignin extraction during isolation process. Another reason for this phenomenon might be higher content of carbohydrate in FMWL. Previous investigation showed that the carbohydrate chains linked to lignin could increase lignin hydrodynamic volume and thereby increase the molar mass of the lignin when it is determined using GPC [18]. This was consistent with the results of sugar analysis as revealed in Table 1.

### 2.5. Thermal Analysis

Thermal gravimetric (TG) analysis is a convenient method to clarify the potential relationship between the thermal properties and chemical structure of lignin. Thus, the thermal stability of the SMWL and FMWL was comparatively investigated by TG analysis and first-derivative thermogravimetric analysis (DTG) thermograms. The TG and DTG curves of these two lignin fractions are shown in Figure S5. As shown, three mass loss steps can be recognized during the thermogravimetric processing of SMWL and FMWL. The TG curves show the first step is below 120 °C because of the evaporation of bound and absorbed water. The primary degradation occurring at 12–500 °C can be attributed to the decomposition of the chemical linkages and carbohydrate components in the lignin fractions into most part of degradation compounds [19]. At the last stage, when the temperature was above 500 °C, DTG covers of FMWL and SMWL showed lower degradation rate. The main volatile compounds products produced from lignin were alcohols, aldehyde acids, and phenolics due to the branched characteristics and aromatic structure of lignin [20]. As shown in Figure S5 of the Supplementary Materials, the maximum degradation rates of SMWL and FMWL were found at 297 and 281 °C, respectively, revealing the lower thermal stability of FWML as compared with SMWL. It is noteworthy that the thermal degradation rate of FMWL was slightly higher as compared with SMWL after the temperature rose above 440 °C. The explanation for this phenomenon might be due to that the higher amount of OCH_3_ groups in FMWL was degraded at the temperature range from 400 to 550 °C [21]. When the temperature reached 640 °C, approximately 42% of residue (42.6% and 40.8% for SMWL and FMWL fractions, respectively) were found, which was similar to the case in the decomposition of lignin fraction of bamboo [22,23]. The results, as depicted in the TG and DTG curves, indicated that the thermal stability of SMWL is better than FMWL.

### 2.6. Pyrolysis-GC/MS Analysis

Pyrolysis-GC/MS analysis was carried out to predict the structural features of FMWL and SMWL fractions. The pyrograms of FMWL and SMWL are graphically presented in Figure S6. Table S1 summarizes the products of lignin pyrolysis by their mass fragments, and the detected lignin-degradation products are listed in Figure S6. As shown, the product distributions of FMWL and SMWL pyrolysis were very complex, and phenol compounds were observed to be most abundant. The identified phenol compounds could be grouped in five categories based on their aromatic substituent groups: (i) aromatic hydrocarbons (AH), mainly benzene, toluene, benzofuran, 1,2,3-trimethylbenzene, and 7-methylbenzofuran; (ii) phenol-type compounds (H), such as 3-ethylphenol, 2,3-dimethylphenol, 2-methylphenol, phenol, and 5-*tert*-butylpyrogallol; (iii) the guaiacol type compounds (G), including vanillin, creosol, 2-methoxy-4-vinylphenol, 4-ethyl-2-methoxyphenol, and 4-hydroxy-3-methoxybenzoic acid. (iv) the catechol type compounds (C), including 4-methyl-1,2-benzenediol, 3-methoxy-1,2-benzenediol, 3-methyl-1,2-benzenediol, and catechol. These compounds mainly result from the *p*-hydroxyphenyl (H), syringyl (S-type), and guaiacyl (G-type) units present in the lignin structure.

For benzene and its derivatives (AH), FMWL showed higher yield (15.0%) as compared with SMWL (10.4%). Toluene, an important variant of benzene-type compounds, was mainly produced from the degradation of the anisole intermediate. The identified phenolic compounds showed that the abundance of G-type compounds was the highest as compared with H-type compounds and S-type compounds. Moreover, high contents of 2-methoxy-4-vinylphenol were released from the lignin fractions. This result might demonstrate the presence of hydroxycinnamic acid derivatives or ferulates in the two lignin fractions [24]. Structural analysis by Py-GC/MS indicated that FMWL and SMWL primarily contained guaiacyl units, thus the G-type compounds comprised the majority of the total pyrolysis products. The formation of G-type compounds as the major products can be attributed to the direct breaking of β-*O*-4 bond which has a lower dissociation energy (55–65 kcal mol^−1^) as compared with that of C–C bonds present in β-5 and 5-5 linkages in lignin (>100 kcal mol^−1^) [25]. It is well known that S-lignin units are mainly involved in β-*O*-4 bond. Obviously, the reason is that an important amount of G-type compounds are generated from S-type compounds by demethoxylation reactions that happen at high temperature. It is worth noting that the total content of C-type compounds was higher than that of H-type compounds. The H and C-type compounds were formed by the breakage of Ar–OCH_3_ and ArO–CH_3_ at high temperatures (600–700 °C), respectively [26]. C-type compounds usually exhibit higher yields as compared with P-type compounds because that the breakage of Ar–OCH_3_ has a higher energy barrier as compared with ArO–CH_3_ [27].

### 2.7. NMR Spectra Analysis

#### 2.7.1. 2D NMR Spectra Analysis

**Figure 2:**
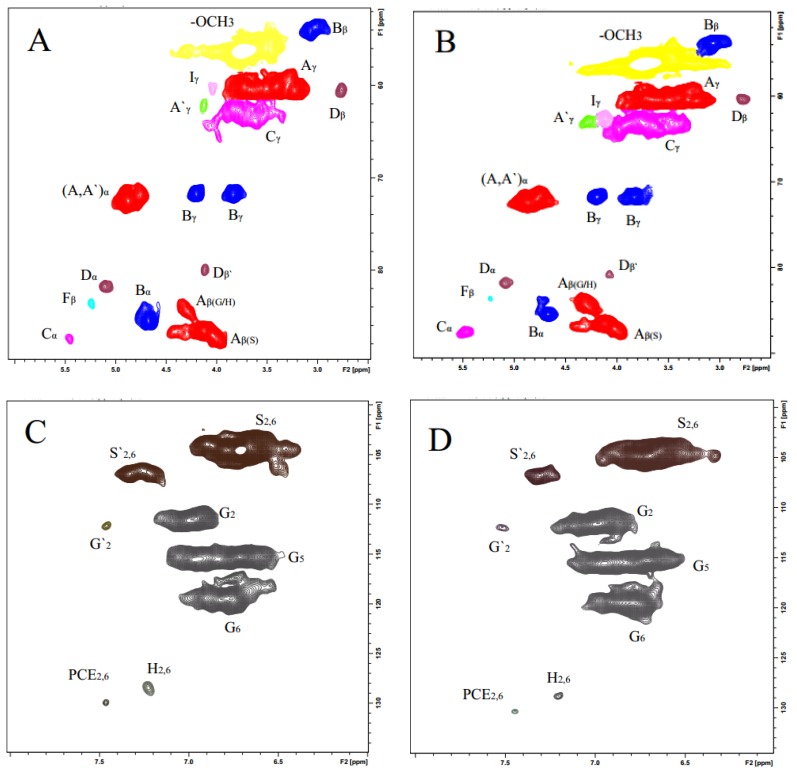
Side-chain (**A**,**B**) and aromatic regions (**C**,**D**) in the 2D HSQC NMR spectra: δ_C_/δ_H_ 50–90/2.5–6.0 and δ_C_/δ_H_ 100–135/6.0–8.0, respectively. (**A**,**C**) FMWL; (**B**,**D**) SMWL. Symbols are taken from Figure 3. See Table 2 for signal assignment.

**Figure 3:**
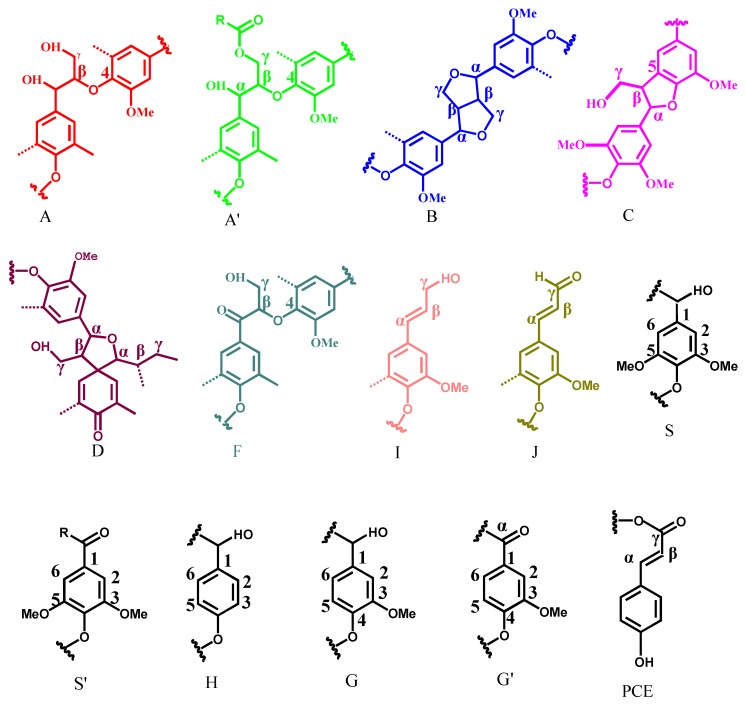
Main substructures of FMWL and SMWL involving various side-chain linkages, and aromatic units identified by 2D HSQC NMR: (**A**) β-*O*-4′ aryl ether linkages with a free-OH at the γ-carbon; (**A**′) β-*O*-4′ aryl-ether linkages with acetylated and/or *p*-hydroxybenzoated-OH at γ-carbon; (**B**) resinol structures formed by β-β′/α-*O*-γ′/γ-*O*-α′ linkages; (**C**) phenylcoumaran structures fromed by β-5′/α-*O*-4′ linkages; (**D**) cinnamate unit; (**F**) C_α_-oxidized β-*O*-4′ substructures; (**G**) guaiacyl unit; (**G′**) oxidized guaiacyl units with a α-ketone; (**S**) syringyl unit; (**S′**) oxidized syringyl unit with a carbonyl group at Cα ketone; (**H**) *p*-hydroxyphenyl unit; (**I**) cinnamyl alcohol end groups; (**J**) cinnamaldehyde end groups; (**PCE**) *p*-coumarate substructures.

2D HSQC NMR is an important tool for investigation of lignin structure because it can provide significant information about the whole macromolecule. In this investigation, the two lignin fractions were investigated by 2D HSQC NMR techniques to understand their structure, particularly the chemical linkages. The 2D HSQC NMR spectra of FMWL and SMWL fractions are shown in Figure 2. On the basis of previous investigations [10,28], the peak assignments in the spectra are annotated (Table 2) and the identified substructures are listed in Figure 3 [29,30,31,32,33,34]. As shown in Figure 2A,B, the side-chain regions (δ_C_/δ_H_ 50–90/2.5–6.0) of FMWL and SMWL in the HSQC spectra appear similar. The obvious signals corresponding to methoxyls (δ_C_/δ_H_ 56.2/3.75) and β-O-4′ aryl ether linkages are observed in both spectra. The β-*O*-4′ substructures (A) were found for α- and γ-C positions at δ_C_/δ_H_ 72.4/4.88, 60.3/3.40–3.70 ppm, and for β-C positions at δ_C_/δ_H_ 86.5/4.13 ppm in S type lignin, and 83.5/4.31 ppm in G and H type lignins. Besides, the signal at δ_C_/δ_H_ 83.5/5.21 ppm is present in both FMWL and SMWL HSQC spectra, which corresponds to the C_β_-H_β_ correlations in oxidized (C_α_=O) β-O-4′ substructures F. The C_γ_–H_γ_ correlations in γ-acylated lignin units (A′) are present at δ_C_/δ_H_ 63.5/4.34–4.46. Resinol (β-β′, B) characterized by their C-H correlations for α-, β- and the double γ-C positions at δ_C_/δ_H_ 85.3/4.68, 54.0/3.07, and 71.9/4.19 and 3.84, respectively. Phenyl coumaran substructures (C) were found for α-, β- and γ-C positions at δ_C_/δ_H_ 87.2/5.50, 53.8/3.33, and 62.9/3.76 ppm. Finally, the C_γ_-H_γ_ correlations in *p*-hydroxycinnamyl alcohol end groups (substructures I) were found at δ_C_/δ_H_ 61.3/4.27 in both FMWL and SMWL HSQC spectra. The substructures D were identified with their C_α_-H_α_ and C_β_-H_β_ correcations at δ_C_/δ_H_ 81.7/5.08, 60.1/2.79 (not shown in Figure 2B), respectively.

The main cross-signals in the aromatic region (δ_C_/δ_H_ 100–135/5.5–8.5) of the 2D NMR spectra of FMWL and SMWL correspond to certain substructures and various aromatic rings in lignin such as syringyl (S), guaiacyl (G), and *p*-hydroxyphenyl (H) units. The S units characterized by C_2,6_–H_2,6_ correlations at δ_C_/δ_H_ 104.7/6.71 ppm, whereas the C_2,6_–H_2,6_ correlations in C_α_=O type S units (δ_C_/δ_H_ 106.9/7.31 ppm) are present in the HSQC spectrum. The G units showed various correlations for C_6_–H_6_, C_5_–H_5_, and C_2_–H_2_ at δ_C_/δ_H_ 119.8/6.79, 115.4/6.71, and 111.7/6.95, respectively. The correlations for the C_6_–H_6_ and C_2_–H_2_ in oxidized α-ketone structures G′ were found at δ_C_/δ_H_ 124.4/7.60 and 112.0/7.51, respectively.

A considerable amount of *p*-hydroxyphenyl (H) units was observed from correlations for C_2,6_-H_2,6_ at δ_C_/δ_H_ 127.6/7.21 ppm, but the C_3,5_–H_3,5_ position correlations overlapped with those from guaiacyl 5-positions. Other signals were also present and assigned to *p*-hydroxycinnamyl alcohol end groups (I), *p*-coumarate substructures (PCE), and cinnamaldehyde end groups (J). The *p*-hydroxycinnamyl alcohol end group (I) was observed for α- and β-C positions at δ_C_/δ_H_ 129.8/6.22 and 129.5/6.41 ppm, respectively. The C_2,6_–H_2,6_ correlations of PCE were found as a weak signal at δ_C_/δ_H_ 130.2/7.48 ppm. The signals for the C_α_–H_α_ and C_β_–H_β_ correlations of substructures J were observed at δ_C_/δ_H_ 154.0/7.59 and 126.5/6.80 (not shown) ppm, respectively.

#### 2.7.2. Quantitative 2D NMR Spectra Analysis

The relative abundances of the basic composition and those of the main linkages were calculated by the 2D HSQC spectra of the SMWL and FMWL fractions based on a previous publication [34,35], and the results are shown in Table 3. As shown, these data indicated an important different composition between SMWL and FMWL fractions. Clearly, the main lignin substructure in SMWL and FMWL fractions was β-*O*-4′ aryl ethers, which accounted for up to 88.1–89.7% of all interunit linkages, while low amounts of β-β′, β-5′ and spirodienones were also found. Comparatively, the FMWL fraction showed a higher β-*O*-4′ aryl ethers as compared with that of SMWL fraction. The S/G ratio, obtained according to the formula S/G = 0.5 × S_2,6_ Integration/G_2_ integration by Wen et al. [36], was important to reveal the top-chemistry of the lignin isolation. In the present investigation, it was observed that FMWL (2.94) fraction had higher S/G ratio than SMWL (1.17) fraction.

#### 2.7.3. ^31^P-NMR Spectra Analysis

**Figure 4:**
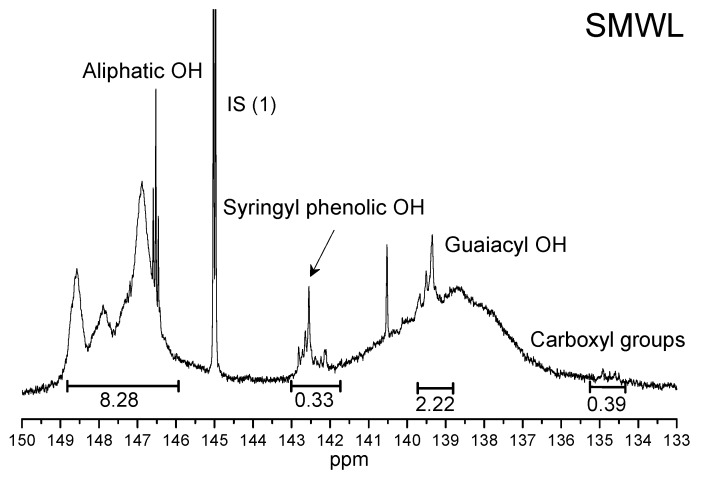

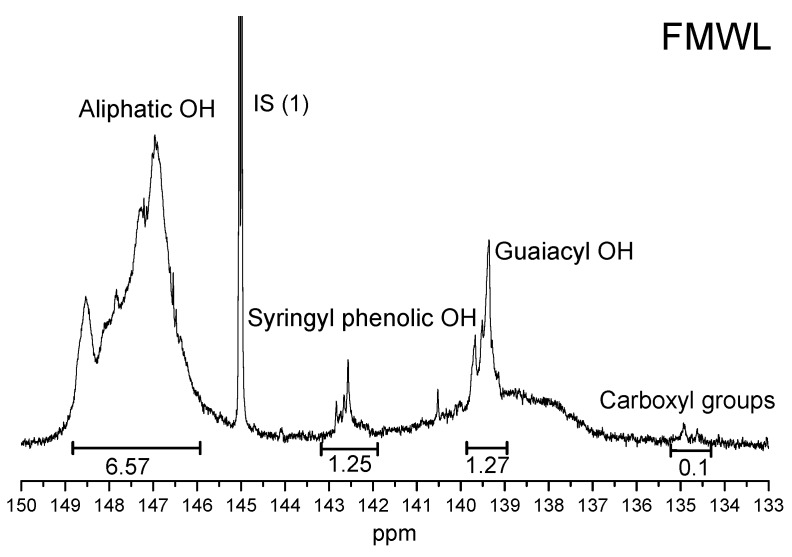
Quantitative ^31^P-NMR spectra of FMWL and SMWL fractions.

To further characterize the functional groups of the two MWL fractions, the two lignin samples were phosphitylated and analyzed by quantitative ^31^P-NMR using cyclohexanol as an internal standard. The spectra are shown in Figure 4.

The concentration of each hydroxyl functional group (in mmol/g) was determined based on the hydroxyl content of the internal standard and its integrated peak area. As shown in Figure 4, the peaks of carboxyl groups (COOH) and hydroxyl (OH) in the two MWL fractions were detected at 134.4–135.0 and 146.0–149.0 ppm, respectively. The peaks of syringyl and guaiacyl phenolic OH groups were observed at 142.3–143.0 and 138.8–140.2 ppm, respectively. Clearly, the SMWL fraction has more aliphatic OH groups than the FMWL. For SMWL and FMWL fractions, the amount of S-type OH was less as compared with that of the corresponding G-type OH. In addition, the aforementioned differences between FMWL and SMWL fractions suggested that FMWL contained more S-type units. The contents of COOH in the SMWL fraction were abundant as compared with FMWL fraction.

## 3. Materials and Methods

### 3.1. Materials

The fresh ripe fruits and stalks of Chinese quince were obtained from a local farm in Nanyang city (China). After removing the seeds, the unpeeled fruits were sliced into 3–5 mm slices. The fruit and stalk of Chinese quince were freeze-dried and air-dried, respectively. The dried samples were finely ground and then sieved to obtain the fraction of 20–40 mesh particle size. The soluble fractions were removed by Soxhlet extraction with benzene/ethanol (2:1, *v/v*), and then the insoluble fractions were air-dried. All standard chemicals were reagent or analytical grade.

### 3.2. Preparation of Milled Wood Lignin

The samples prepared after benzene/ethanol extraction were first milled for 6 h in a planetary ball mill (Changsha Tianchuang Powder Technology Co., Ltd., Changsha, China), then extracted using 96% dioxane (*v/v*) and purified based on the published paper [37]. The lignin samples from stalks and fruits were labeled as SMWL and FMWL, respectively.

### 3.3. Characterization

The carbohydrate moieties associated with the two lignin fractions were determined by hydrolysis with dilute sulfuric acid [38]. Sugar analysis (neutral sugars and uronic acids) was conducted by using high performance anion exchange chromatography (HPAEC) [39]. The neutral sugars and uronic acids in the fractions were liberated by hydrolysis with 72% H_2_SO_4_ for 45 min at 25 °C followed by a high temperature hydrolysis at 105 °C for 2.5 h after dilution to 3% H_2_SO_4_. After hydrolysis, the samples were diluted and injected into the HPAEC system (Dionex ISC3000, Sunnyvale, CA, USA) with an amperometric detector, a CarbopacTMPA-20 column (4 × 250 mm, Dionex), and a guard PA-20 column (3 × 30 mm, Dionex). Calibration was performed with standard solutions of l-arabinose, d-glucose, d-xylose, d-mannose, d-galactose, glucuronic acid and galacturonic acid.

The weight-average (Mw) and number-average (Mn) molecular weights of the lignin fractions were determined by gel permeation chromatography (GPC, Agilent 1200, Lexington, MA, USA) with a refraction index detector (RID) on a PL-gel 10 mm MixedB 7.5 mm ID column, calibrated with monodisperse polystyrene. The samples were acetylated with acetic anhydride/pyridine (1:1, *v/v*) before determination. 200 mg sample was dissolved in 8 mL acetic anhydride/pyridine and stirred for 24 h in darkness. After drying, 2 mg of the acetylated sample was dissolved in 2 mL tetrahydrofuran, and 20 μL sample in solution was injected. The column was operated at ambient temperature and eluted with tetrahydrofuran at a flow rate of 1 mL/min.

Pyrolysis-gas chromatography/mass spectrometric (Py-GC/MS) analysis was carried out using a CDS 5250 pyrolyzer coupled with an Agilent GC/MS system (6890N GC, 5973 mass detector). In each experiment, 0.5 mg lignin was placed in a quartz filler tube. Helium (99.999%) was used as the carrier gas with a constant flow rate of 1 mL min^−1^ and a 1:80 split ratio. The pyrolysis temperature was carried out at 300 °C for 5 s, then at the rate of 20 °C/min up to 700 °C and hold for 5 s. The pyrolysis volatiles were analyzed online by GC/MS. And the transfer line and injector temperature were kept at 300 °C. The chromatographic separation was performed using a TR-5MS capillary column (30 m × 0.25 mm i.d., 0.25 μm film thickness). The oven temperature was programmed form 40 °C for 3 min, then increased to 230 °C at a rate of 20 °C/min, then up to 280 °C at a rate of 20 °C/min and held for 15 min at 270 °C. The mass spectrometer was operated in EI mode at 70 eV. The mass spectra were obtained from *m/z* 30 to 500 with the scan rate of 500 amu s^−1^. Identification of chromatographic peaks was achieved according to the NIST library and the relevant literature [40,41,42].

FT-IR spectra of the two MWL fractions were obtained on a Nicolet-510-type FT-IR spectrophotometer (ThermoFisher, Lexington, MA, USA). The samples (1%) were mixed and ground with KBr powder, and then pressed into 1 mm pellets. Thirty-two scans were recorded for each MWL fraction in the range of 4000–800 cm^−1^.

Ultraviolet (UV) spectroscopic analysis was performed on a water-dioxane solution (1:9, *v/v*) using a UV 2300 (Shanghai Tianmei Scientific Instrument Co. Ltd., Shanghai, China) in a 1 cm cells. The sample concentration was 0.001 g/mL, and the absorption coefficients were determined in the range from 190 to 600 nm at 2 nm intervals resulting in 205 points for each sample.

2D Heteronuclear Single Quantum Correlation (HSQC) NMR and ^31^P-NMR experiments of two MWL fractions were performed at 25 °C using an AVIII 400 MHz spectrometer (Bruker, Sunnyvale, CA, USA) [43]. 90 mg of MWL fractions were dissolved in 0.5 mL DMSO-*d*_6_ for 2D-HSQC spectra and 2D-HSQC spectra was recorded in heteronuclear single quantum correlation (HSQC) experiments. The spectral widths were 5000 and 20,000 Hz for the ^1^H and ^13^C-dimensions, respectively. The number of collected complex points was 1024 for 1H-dimension with a recycle delay of 1.5 s. The number of transients was 64, and 256 time increments were always recorded in ^13^C-dimension. The ^1^*J*_CH_ used was 145 Hz. The J-coupling evolution delay was set to 3.2 ms. Squared cosine-bell apodization function was applied in both dimensions. Prior to Fourier transformation, the data matrixes were zero filled up to 1024 points in the ^13^C-dimension. And ^31^P-NMR spectra were acquired afer reacting lignin with 2-chloro-4,4,5,5-tetramethyl- 1,3,2-dioxaphospholanyl chloride. A known amount of dry lignin (20 mg) was dissolved in anhydrous pyridine (500 μL) and deuterated chloroform (1.6:1, *v/v*) under stirring. This was followed by the addition of cyclohexanol (100 μL, 10.85 mg mL^−1^) as an internal standard (IS), and chromium acetylacetonate solution (100 μL, 5 mg mL^−1^ in anhydrous pyridine and deuterated chloroform 1.6:1, *v/v*) as relaxation reagent. Finally, the mixture was treated with phosphorylating reagent (100 μL, 2-chloro-4,4,5,5-tetramethyl-1,3,2-dioxaphospholane) and was transferred into a 5 mm NMR tube for analysis. After the reagent was added, the tube was closed and shaken for a few minutes to ensure proper mixing during the reaction. Relaxation delay was set to 5. Approximately 1000 transients were acquired to ensure a high signal/noise ratio. For each lignin sample at least two spectra were recorded to decrease the standard error. Data processing was performed using standard Bruker Topspin-NMR software (Bruker Corp., Billerica, MA, USA).

The changes in stalk and fruit morphology of Chinese quince before and after milling were observed by scanning electron microscopy (SEM). For magnification, each sample was viewed at an accelerating voltage of 30 kV.

Thermal properties of the two MWL samples were evaluated using thermogravimetric (TG) and differential thermogravimetric (DTG) on a DTG-60 type of simultaneous thermal analyzer (DTG-60, Shimadzu, Japan). 8–10 mg lignin samples were fed into an aluminum crucible and heated from 30 to 640 °C at a heating rate of 10 °C/min in a nitrogen atmosphere.

## 4. Conclusions

The chemical structure properties of the lignins in Chinese quince fruit and stalk were comparatively investigated. The results showed that the lignin fractions in Chinese quince fruit and stalk were mainly composed of β-*O*-4 ether bonds and β-5 and β-β′ carbon-carbon linkages; however, significant differences were found in S/G ratio and carbohydrate linkages in the two lignin fractions. Compared to the SMWL, the FMWL fraction had a higher S/G ratio and more carbohydrate linkages. The predominant carbohydrates associated with FMWL and SMWL fractions were cellulose and xylan-type hemicelluloses, respectively. In addition, the lignin fraction isolated from stalk of Chinese quince exhibited a slightly higher thermal stability as compared to the FMWL fraction. It is believed that the understanding the structure features of lignin in Chinese quince fruits will contribute to give insight to the properties of lignin and enable the food processing industry to separate lignin more efficiently. Further study should be focused on the structure-activity relationships of the lignin fractions from Chinese quince fruits and stalks.

## Figures and Tables

**Table 1:** Yield and Carbohydrate Composition of SMWL and FMWL Fractions (% dry weight).

Sample	Yield of MWL (%)	Total Sugar Content (%)	Carbohydrate Composition Content (Based on Total Sugar, %)					
			Gal ^a^	Glu ^a^	Man ^a^	Ara ^a^	Xyl ^a^	Glua ^a^
SMWL	7.3	0.67	13.4	20.9	-	14.9	38.8	11.9
FMWL	0.8	1.39	18.7	58.3	15.8	7.2	-	-

^a^ Gal, galactose; Glu, glucose; Man, manose; Ara, arabinose; Xyl, xylose; Glua, gluconic acid.

**Table 2:** Assignment of Main Lignin ^13^C-^1^H Cross-signals in the HSQC Spectra of the FMWL and SMWL Fractions.

Label	δ_C_/δ_H_	Assignment
OCH_3_	56.2/3.75	C-H in methoxyls
(A, A′)_α_	72.4/4.88	C_α_-H_α_ in β-*O*-4′ substructures (A) and γ-acylated β-*O*-4′ substructures (A′)
A_β_ (G/H)	84.5/4.31	C_β_-H_β_ in β-*O*-4′ substructures linked to G and H units (A)
A_β_ (S)	86.5/4.13	C_β_-H_β_ in γ-acylated β-*O*-4′ substructures linked to S units (A′)
A_γ_	60.3/3.40–3.70	C_γ_-H_γ_ in β-*O*-4′ substructures units (A)
A′_γ_	63.5/4.34–4.46	C_γ_-H_γ_ in γ-acylated β-*O*-4′ substructures units (A′)
B_α_	85.3/4.68	C_α_-H_α_ in resinol substructures (B)
B_β_	54.0/3.07	C_β_-H_β_ in resinol substructures (B)
B_γ_	71.9/3.84 and 4.19	C_γ_-H_γ_ in resinol substructures (B)
C_α_	87.2/5.50	C_α_-H_α_ in phenylcoumaran substructures (C)
C_β_	53.8/3.33	C_β_-H_β_ in phenylcoumaran substructures (C)
C_γ_	62.9/3.76	C_γ_-H_γ_ in phenylcoumaran substructures (C)
D_α_	81.7/5.08	C_α_-H_α_ in spirodienone substructures (D)
D_β_	60.1/2.79	C_β_-H_β_ in spirodienone substructures (D)
F_β_	83.5/5.21	C_β_-H_β_ in oxidized (C_α_=O) β-*O*-4′ substructures (F)
I_γ_	61.3/4.27	C_γ_-H_γ_ in *p*-hydroxycinnamyl alcohol end groups (I)
FA_β_	113.9/6.29	C_β_-H_β_ in cinnamate unit
G_2_	111.7/6.95	C_2_-H_2_ in guaiacyl units (G)
G_6_	119.8/6.79	C_6_-H_6_ in guaiacyl units (G)
G_5_	115.4/6.71	C_5_-H_5_ in guaiacyl units (G)
G′_2_	112.0/7.51	C_2_-H_2_ in oxidized (C_α_=O) guaiacyl units (G′)
G′_6_	124.4/7.60	C_6_-H_6_ in oxidized (C_α_=O) guaiacyl units (G′)
S_2,6_	104.7/6.71	C_2,6_-H_2,6_ in etherified syringyl units (S)
S′_2,6_	106.9/7.31	C_2,6_-H_2,6_ in oxidized (C_α_=O) syringyl units (S′)
H_2,6_	128.7/7.21	C_2,6_-H_2,6_ in *p*-hydroxyphenyl units (H)
J_α_	154.0/7.59	C_α_-H_α_ in cinnamaldehyde end groups (J)
J_β_	126.5/6.80	C_β_-H_β_ in cinnamaldehyde end groups (J)
I_α_	129.8/6.22	C_α_-H_α_ in *p*-hydroxycinnamyl alcohol end groups (I)
I_β_	129.5/6.41	C_β_-H_β_ in *p*-hydroxycinnamyl alcohol end groups (I)
PCE2,6	130.2/7.48	C_2,6_-H_2,6_ in *p*-coumarate (PCE)

**Table 3:** Quantification of the FWML and SMWL by quantitative 2D HSQC method.

Lignin Interunit Linkages	Percentage (%)	
	SMWL	FMWL
β-*O*-4-Aryl ethers (A/A′)	88.10	89.70
β-β (Resinols) (B)	9.80	8.30
β-5 (Phenylcoumarans) (C)	0.70	1.50
Spirodienones (D)	1.40	0.50
Syringyl Units (S_2,6_)	53.68	74.07
Guaiacyl Units (G_2_)	46.00	25.19
*p*-Hydroxyphenyl units (H_2,6_)	0.32	0.74
S/G ratio	1.17	2.94

## Supplementary Materials

The following are available online at www.mdpi.com/1420-3049/22/6/890/s1, Table S1: Identity and relative molar abundances of the compounds released after Py-GC/MS of the FMWL and SMWL, Figure S1: The structure of compounds in Table S1; Figure S2: FT-IR spectra of two ball-milled lignins of Chinese quince stalk and fruit; Figure S3: UV spectra of FMWL and SMWL; Figure S4: SEM of Chinese quince stalk and fruit before and after milling; Figure S5: Thermograms of SMWL (curve a) and FMWL (curve b); Figure S6: Pyrograms of Py-GC/MS of FMWL and SMWL.
