# Supplementary material for: Structural Characterization of Lignin in Fruits and Stalks of Chinese Quince
Source: Molecules. 2017 May 27;22(6):890. doi: 10.3390/molecules22060890 (PMC6152639; doi:10.3390/molecules22060890)
Supplement: Supplementary file 1 [file molecules-22-00890-s001.pdf]

Supplementary Materials: Structural characterization of lignin in fruits and stalks of Chinese quince

Hui-Shuang Yin, Hua-Min Liu* and Yu-Lan Liu

Table S1. Identity and relative molar abundances of the compounds released after Py-GC/MS of the FMWL and SMWL.

Label	R.T. (min)	Compound	Type	FMWL (%)	SMWL (%)
1	3.18	Benzene	AH	2	2
2	4.56	Toluene	AH	2.8	1.9
3	5.92	Benzene, 1,3-dimethyl-	AH	0.6	0.4
4	6.98	Benzaldehyde	AH	0.5	0.9
5	7.08	Phenol	H	2.4	1.8
6	7.30	Benzene, 2-propenyl-	AH	0.7	
7	7.33	Benzofuran	AH	1	0.8
8	7.57	Benzene, 1,2,3-trimethyl-	AH	0.6	
9	7.67	Benzyl alcohol	H		0.6
10	7.80	Phenol, 2-methyl-	H	1.8	2
11	7.99	p-Cresol	H	3.1	2.1
12	8.14	Phenol, 2-methoxy-	G	3	3.5
13	8.27	Benzofuran, 7-methyl-	AH	0.3	0.7
14	8.33	Benzofuran, 2-methyl-	AH	0.5	0.2
15	8.63	Phenol, 2,3-dimethyl-	H	0.7	0.8
16	8.77	Phenol, 3-ethyl-	H	1.6	
17	8.79	3-Hydroxy-2-methylbenzaldehyde	H		0. 7
18	8.89	Phenol, 2-methoxy-3-methyl-	G		0.5
19	8.97	Catechol	C	9.8	5.2

20	9.00	Creosol	G	2.2	2.8
21	9.06	Naphthalene	AH	1.2	1.1
22	9.12	Phenol, 2,4,5-trimethyl-	H		0.7
23	9.26	Benzaldehyde, 3,4-dimethyl-	AH	0.9	
24	9.48	1,2-Benzenediol, 3-methyl-	C	0.9	1
25	9.53	1,2-Benzenediol, 3-methoxy-	C	1	1.8
26	9.65	Phenol, 4-ethyl-2-methoxy-	G	1.8	2.3
27	9.69	1,2-Benzenediol, 4-methyl-	C	3.5	1.2
28	9.77	1H-Inden-1-one, 2,3-dihydro-	AH	1.7	1.1
29	9.80	Cinnamaldehyde, .beta.-methyl-	AH		0.5
30	9.93	Phenol, 2-methoxy-4-vinyl-	G	4.3	5.1
31	10.04	1-Methylindan-2-one	AH	1.3	0.8
32	10.16	Phenol, 2,6-dimethoxy-	S	3.7	7.8
33	10.38	1, 1, 5-Trimethyl-1, 2-dihydronaphthalene	AH	0.9	
34	10.54	Vanillin	G		2
35	10.79	Benzoic acid, 4-hydroxy-3-methoxy-	G		5.5
36	10.81	1,2,3-Trimethoxybenzene	S	2.6	
37	10.87	trans-Isoeugenol	G	1.3	1.7
38	11.20	Phenol, 2,4-bis(1,1-dimethylethyl)-	H	0.8	0.5
39	11.30	5-tert-Butylpyrogallol	C	2	4.4
40	11.36	Homovanillic acid	G		0.4
41	11.56	2,6-Dimethyl-3-(methoxymethyl)-p-benzoquinone	H	2.4	6
42	11.77	Phenol, 2,6-dimethoxy-4-(2-propenyl)-	S	0.3	0.7
43	12.14	Benzaldehyde, 4-hydroxy-3,5-dimethoxy-	S	0.8	2. 4

44	12.56	Ethanone, 1-(4-hydroxy-3,5-dimethoxyphenyl)-	S	0. 9	2.5
		Five categories	AH%	15	10.4
			H%	12.8	14.5
			G%	12.6	23.8
			S%	7.4	11
			C%	17.2	13.6

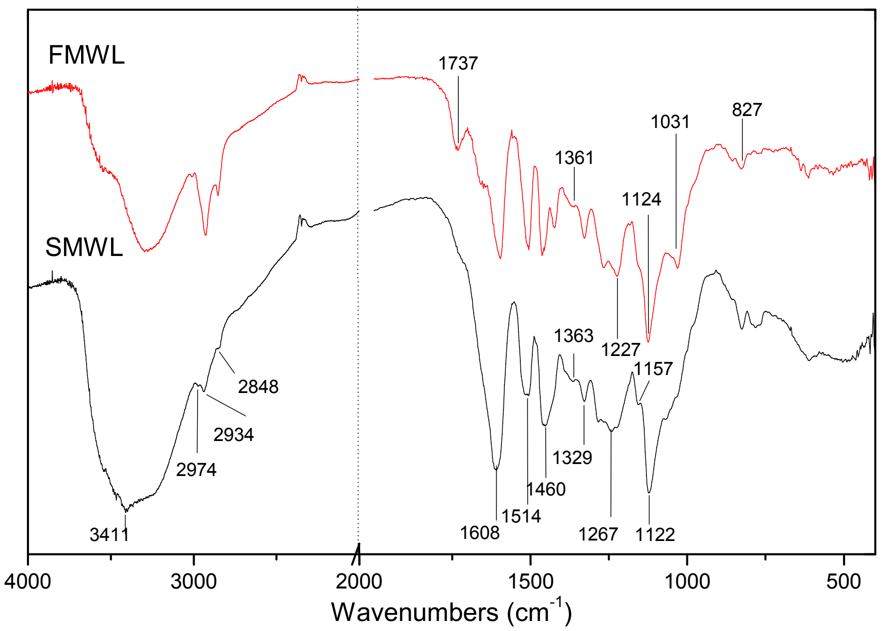

Figure S1. FT-IR spectra of two ball-milled lignins of Chinese quince stalk and fruit.

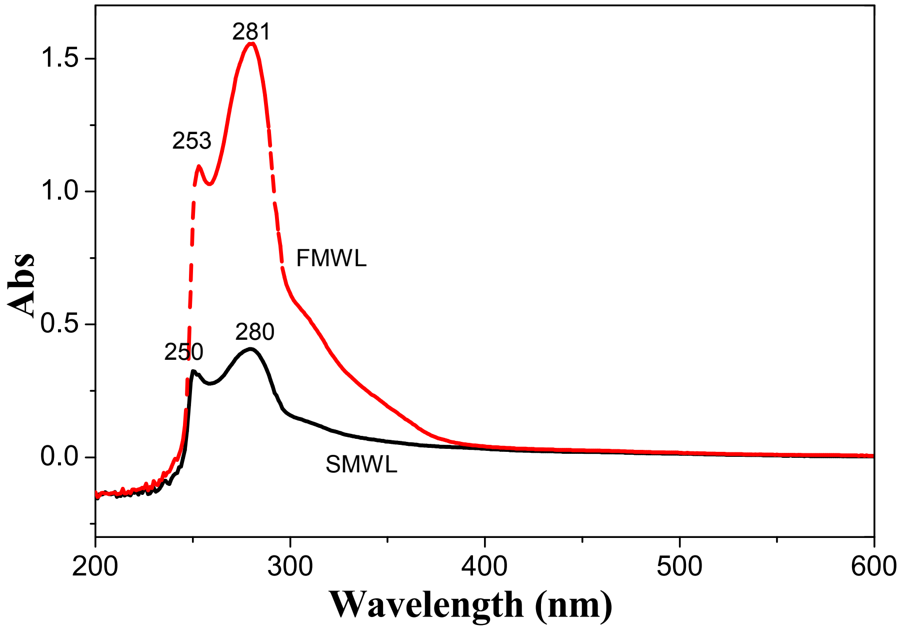

Figure S2. UV spectra of FMWL and SMWL.

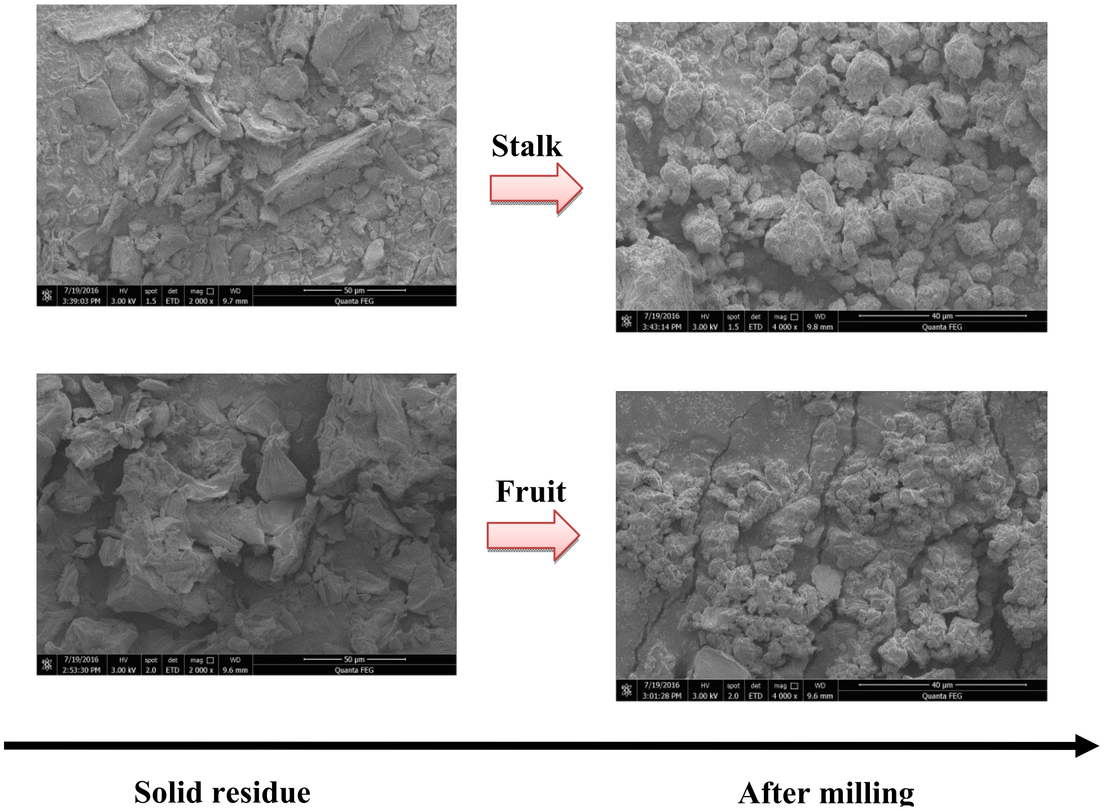

Figure S3. SEM of Chinese quince stalk and fruit before and after milling.

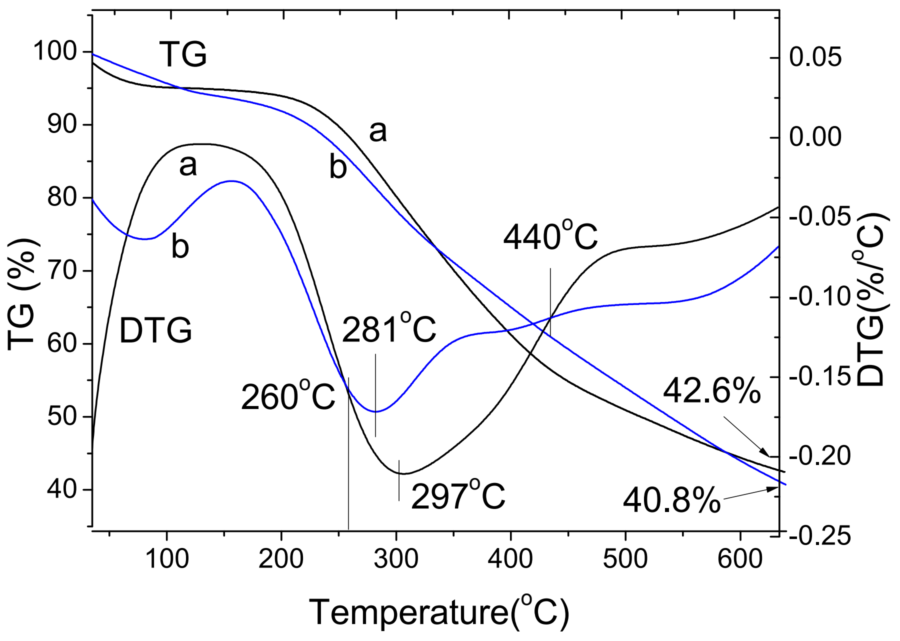

Figure S4. Thermograms of SMWL (curve a) and FMWL (curve b).

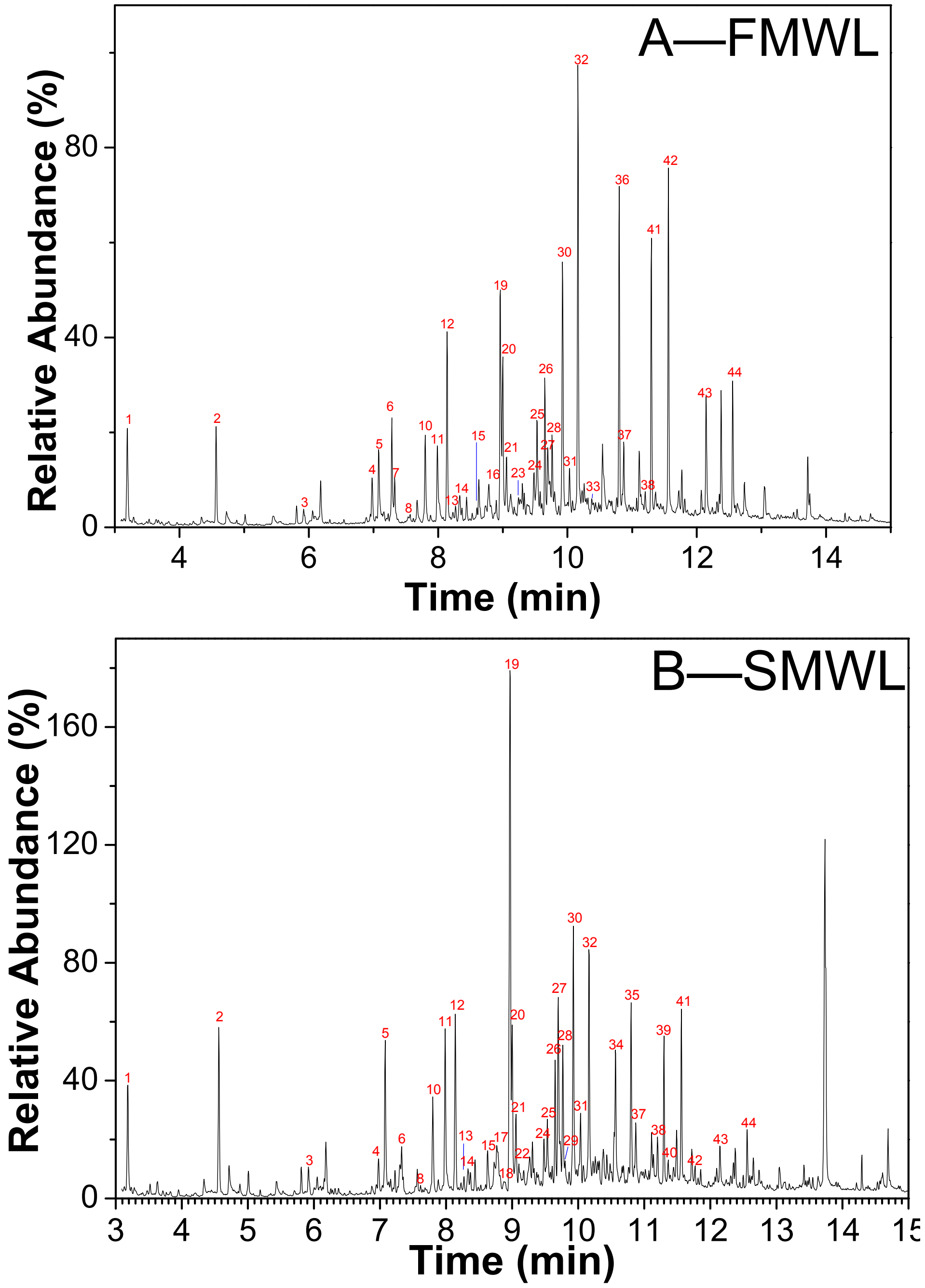

Figure S5. Pyrogram of Py-GC/MS of FMWL and SMWL.

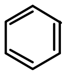
3.18

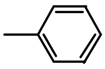
4.56

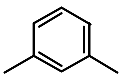
5.92

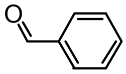

6.98

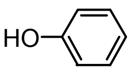

7.08

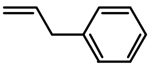
7.30

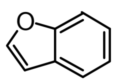

7.33

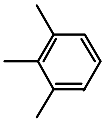
7.57

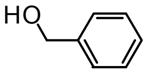

7.67

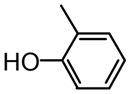

7.80

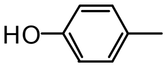

7.99

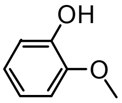

8.14

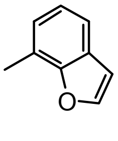

8.27

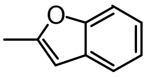

8.33

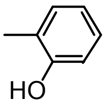

8.63

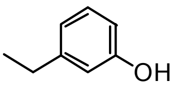

8.77

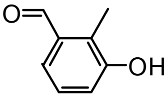

8.79

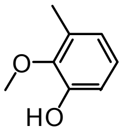

8.89

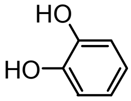

8.97

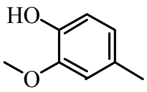

9.00

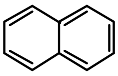
9.06

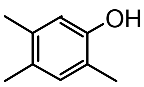

9.12

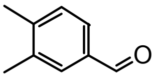

9.26

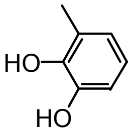

9.48

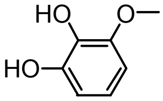

9.53

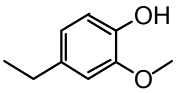

9.65

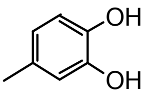

9.69

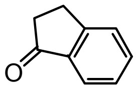

9.77

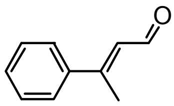

9.80

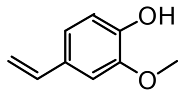

9.93

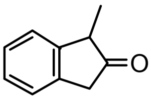

10.04

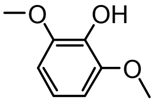

10.16

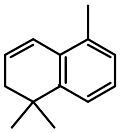
10.38

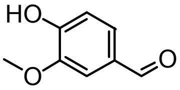

10.54

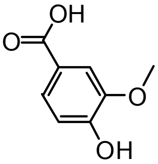

10.79

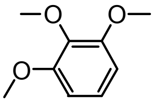

10.81

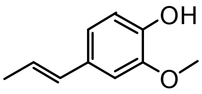

10.87

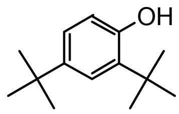

11.20

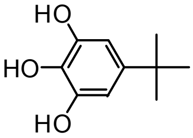

11.30

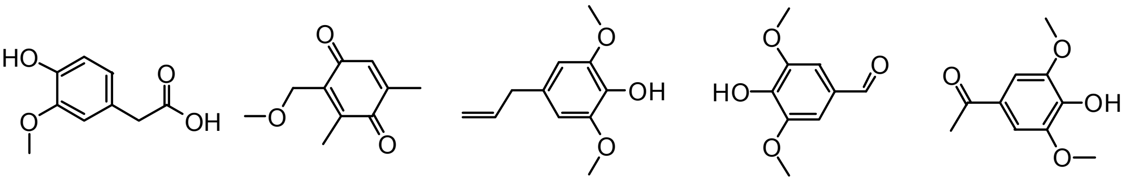

11.36 11.56 11.77 12.14 12.56

Figure S6. The structure of compounds in table S1.
